# Association between factors related to the pregnancy, neonatal period, and later complications (especially asthma) and menarcheal age in a sample of Lebanese girls

**DOI:** 10.1186/s12905-020-01101-7

**Published:** 2020-10-16

**Authors:** Josephine Sakkal, Souheil Hallit, Georges Nicolas

**Affiliations:** 1 Faculty of Medicine and Medical Sciences, Holy Spirit University of Kaslik (USEK), Jounieh, Lebanon; 2 Department of Pediatrics, Notre Dame des Secours University Hospital, Byblos, Lebanon; 3 INSPECT-LB: Institut National de Santé Publique, Épidémiologie Clinique et Toxicologie- Liban, Beirut, Lebanon

**Keywords:** Menarcheal age, NICU admission, Gestational age, Phototherapy, Preeclampsia, Asthma

## Abstract

**Background:**

Studies about the majority of the factors that may potentially influence the pubertal timing and menarche were controversial. The objective was to evaluate the association between factors related to the pregnancy, neonatal period, and the complications that may happen later in life and the menarcheal age in a sample of Lebanese girls admitted or not to the NICU at birth. Our secondary objective was to try to find, for the first time in literature, a correlation between respiratory distress at birth and the need of oxygen therapy with the age of the first menses in these girls.

**Methods:**

It is a cross-sectional retrospective study, conducted between January and March 2019. Our sample included all the 2474 girls born in Notre-Dame-de-Secours hospital, between 2000 and 2005; the sample consisted of 297 girls (97 girls admitted to the NICU and 200 randomly chosen to participate in our study with a ratio of 1:2 (1 girl admitted to the NICU vs 2 girls born in the nursery).

**Results:**

Asthma later in life was significantly associated with lower age at menarche in girls, whereas a higher mother’s age at menarche and a higher gestational age were significantly associated with higher age at menarche in girls. When taking each cause of NICU admission as an independent variable, showed that a higher mother’s age at menarche was significantly associated with higher age at menarche in girls, whereas a higher number of days of phototherapy, a preeclampsia in the mother during pregnancy and asthma later in life in the girl were significantly associated with a lower age at menarche in girls.

**Conclusion:**

The timing of menarche seems to be associated with many factors in Lebanese girls that should not be disregarded by physicians.

## Background

Physiological age of puberty is influenced by many nutritional, metabolic, genetic and environmental factors [1, 2]. During the last century, a trend toward an earlier puberty and menarcheal age was noticed in girls [1, 3]. Many authors associated precocious puberty, which is defined by the appearance of the secondary sexual characteristics before the age of 8 years in girls [3, 4], to the development of many diseases later on [4]. These include a higher risk of development of cardiovascular, oncologic, gynecologic, obstetric, gastrointestinal, musculoskeletal and neurocognitive diseases during adolescence and adulthood according to some studies [4]. Specifically, early menarche was associated with elevated psychopathology that may or not persist during adulthood, including depression and antisocial behaviors, disrupting emotional, social and even academic paths [5, 6]. Several studies revealed a relationship between pubertal timing and many other factors related to the pregnancy, neonatal period, and the complications that may happen later in life.

To start with the fetal period, many researchers showed that girls born from mothers who were overweight during pregnancy and lactation are at a higher risk to manifest an intrauterine growth restriction, and to present an alteration in their metabolic and endocrine functions [7]; this modifies their reproductive system, leading to a precious thelarche (defined as the appearance of breast tissue as a result of initial estrogen activity) and pubarche later on (the appearance of pubic hair under the effect of androgens) [7, 8]. In addition, malnutrition during pregnancy predisposes the newborn to a growth restriction during the neonatal period, followed by obesity during childhood, leading to an acceleration in the pubertal timing during adolescence [9].

Smoking during pregnancy also influences the menarcheal age; authors agreed that it affects the fetal hypothalamus and endocrine system, putting the fetus at a higher risk of prematurity and growth restriction, with a higher child’s tendency to develop more obesity during childhood and puberty [10, 11]. While some researchers attributed smoking exposure during the antenatal period to a later pubertal age [12], others demonstrated a negative association between smoking exposure and the pubertal and menarcheal age [12–14]. Concerning alcohol during pregnancy, it’s also agreed that it causes an intrauterine growth restriction [15], and disrupts the neuroendocrine regulation in the hypothalamo-pituitary-gonadic axis and pubertal development later on [15–17].

In addition, genetics play a major role in controlling the timing of puberty, although only a very small percentage of these specific genes and trait variants is known so far [18, 19]. This might explain the relationship found between the mothers’ precocious menarcheal age and the manifestation of a precocious thelarche and menarche in their daughters [20, 21]. In fact, studies about this subject were controversial, and some showed that mother’s menarcheal age can only predict the timing of the first menstruations in their non-obese descendants, and that a high BMI in girls before puberty can accelerate the menarcheal timing regardless of their mother’s meanarcheal age [22].

Another factor that may affect the hypothalamic-pituitary-gonadic axis and the endocrine functions of newborns is the administration of glucocorticoids to the mothers during pregnancy, in order to accelerate fetal organs’ maturation, and to reduce the incidence of the neonatal respiratory distress syndrome [23]. Studies showed that their excess may lead to a delay in the timing of puberty, and to a reduction of fertility later on [23, 24].

In addition, authors revealed that preeclampsia during pregnancy is one of the main causes of fetal intrauterine growth restriction, and that this deficient metabolic state may lead to a precocious puberty during adolescence [22, 25], although studies concerning this subject were controversial [22, 25]. During the neonatal period, prematurity may lead to a precocious menarcheal age according to some studies but not others [26, 27]. In addition, a meta-analysis found that puberty appeared earlier in infants born small for gestational age (SGA) than those who were appropriate for gestational age, and that menarche was significantly more precocious in SGA newborns, but within normal ranges [28]. However, other findings didn’t find any difference between the two groups [28]. Furthermore, exclusive breastfeeding was directly related in some studies to a delay in thelarche and menarcheal age, while others couldn’t find any association between breast milk and pubertal age [29, 30].

Finally, concerning the impact of life complications on pubertal timing, neonatal infections and those happening during early childhood seem to delay the pubertal and menarcheal age [31]. In addition, some authors demonstrated that asthma and its treatment with inhaled corticoids delayed the pubertal timing [32, 33], although findings were controversial about this subject [34]. However, Childhood obesity accelerates the age of puberty and menarche [35, 36], noting that premature and SGA infants are at a higher risk to develop obesity later on [37, 38]. Psychosocial factors, such as family disruption, father’s absence and continuous stress also seem to lead to a more precocious sexual maturation and menarcheal age [22], especially if these factors occur during early childhood (before the age of 5 years where girls seem to be the most sensitive to family composition). In addition, adverse experiences during childhood, such as sexual or physical abuse, nervous troubles or alcohol addictions in parents are strongly associated with a precocious sexual maturation [20]. Obesity is another factor linked to early onset in girls according to a meta-analysis [35]. Finally, socioeconomic factors, such as family income, educational level and type of residency also seem to affect the psychosocial status during childhood and adolescence, which accelerates the pubertal development, especially in developing countries [20].

Menarche is an important milestone reached by adolescents. In fact, the experience of menarche affects many aspects of the girls’ lives, including their sexual identification, their perception of their body image and their relations with their family and friends [39]. It is associated with a higher maturity level in adolescents, especially when they are well prepared to this biological milestone, and aware that their reproductive health is on track. In addition, it may offer a chance for girls to bond with one another. However, early maturating and unprepared girls may experience confusion, ambivalence and embarrassment [40]. This highlights the need to explain menarche to girls and prepare them psychologically well before it could possibly occur.

In general, studies showed that mothers are the main source of preparation and knowledge to their girls about menstruation [41]. However, many factors may influence their readiness to share their experience, including social ban and taboos, embarrassment, lack of enough information and sometimes their negative attitude while discussing menstruation [41]. This highlights the importance of training programs for mothers to overcome these restrictions and to strengthen their relationship with their daughters [41, 42].

As described above, studies about the majority of the factors that may potentially influence the pubertal timing and menarche were controversial. In addition, many of these factors may lead to a neonatal intensive care unit (NICU) admission at birth, including neonatal infections [43], prematurity [43] and SGA [44]. Our objective was to evaluate the association between factors related to the pregnancy, neonatal period, and the complications that may happen later in life, especially asthma, and the menarcheal age in a sample of Lebanese girls admitted or not to the NICU at birth.

## Methods

### Study design

**Fig. 1:**
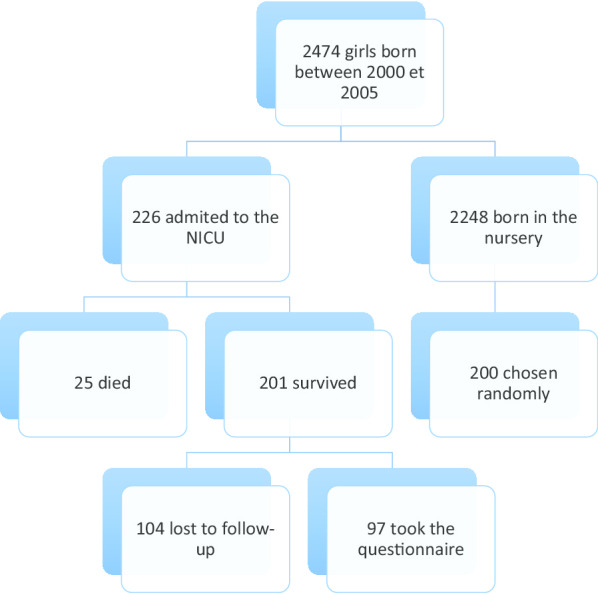
Flow chart of the study design

It is a cross-sectional retrospective study, conducted between January and March 2019. Our sample included all the 2474 girls born in Notre-Dame-de-Secours hospital, between 2000 and 2005 (current age between 14 and 19 years old). These 6 years were chosen since the girls born during this period would have reached by now their menarcheal age most probably. No exclusion criteria were applied. A total of 226 girls was admitted to the NICU during this period, of which 25 didn’t survive the neonatal period; 104 girls were lost to follow-up to the change in the families’ phone numbers during these years, leaving a final number of 97 girls admitted to the NICU, of whom 62 were premature. To compare them to the girls born during the same period without the necessity to be admitted to the NICU, the same procedure was followed. In fact, 2248 girls were born in the nursery between 2000 and 2005, of which 200 were randomly chosen to participate in our study with a ratio of 1:2 (1 girl admitted to the NICU vs 2 girls born in the nursery). The randomization was done using an online software (www.randomizer.org). This final sample consisted of 297 girls (Fig. 1).

### Minimal sample size calculation

According to the G-power software, and based on an effect size f2 = 1.5%, an alpha error of 5%, a power of 80%, and taking into consideration 25 factors to be entered in the multivariable analysis, the results showed that a minimal number of 172 was needed.

### Procedure followed with the girls

The questionnaire developed for this study is provided as Additional File 1. It consisted of 32 questions and was lead over the phone with the enrolled girls, after explaining the target of the study to each of them and taking their oral consent to participate. The neonatal data of all the girls born in the hospital during these 6 years was collected from their medical files (birth date, gestational age, weight, height and head circumference at birth, the major cause of her admission to the NICU if any, the manifestation of a preeclampsia in her mother during pregnancy, the occurrence of a respiratory distress at her birth with the need of oxygen therapy, the mode and days of ventilation, and the necessity to take surfactant or corticoids, the Apgar score at 1 and 5 min, parity, the administration of antibiotics for a neonatal infection, and the exposition and the days of phototherapy if any. A SGA newborn was defined as having a birth weight below the 10th percentile for gestational age, using the 2010 Olsen growth curves [45].

Each girl was questioned about her actual age, weight and height at menarche, her actual weight and height, the regularity of her menstrual cycles, the days of menstruation, and the abundance of bleeding. A regular cycle was defined as having a consistent length between 21 and 35 days, with a maximum of 7 days of changes in the duration of cycles [46, 47]. Girls were also asked about active smoking with the type and quantity of tobacco (cigarettes and number of cigarettes per day, waterpipe and number of waterpipes per week) and about passive smoking with the number of smokers inside the house, and frequency of exposure in cafes and restaurants per week. All data were self-reported and subjective.

Concerning the abundance of bleeding, each girl was asked if she needs to change tampons or pads at an interval of less than 3 h, if she uses more than 21 pads/ tampons per cycle, the necessity to change at night, the presence of big clots during the bleeding period, and if she suffers or not from anemia.

Presence of signs of hyperandrogenism: To identify these girls, a question about hirsutism was asked, and it was classified based on the Ferriman-Gallwey score [40].

Girls were classified as having no hirsutism (score < 8), light hirsutism (score between 8 and 15), moderate (score between 16 and 25) or severe (score > 25) [48]. Girls were also asked about a physician diagnosis of a polycystic ovary syndrome (PCOS), with the necessity of treatment with oral contraceptives and about the presence of any identified pathology during their life, which necessitated the intake of any medication.

### Procedure followed with the girls’ mothers

After taking their oral consent to participate to our study, each mother was questioned over the phone about her actual age, the breastfeeding period if any, her smoking habits during and after pregnancy, with the type of tobacco and its quantity (cigarettes with the number of cigarettes per day, waterpipe with the number of heads per week, cigars with their number per week), alcohol intake during pregnancy, with the type and number of glasses per week, her age at menarche, the presence of any familial history of prematurity and if her daughter developed asthma during childhood.

### Statistical analysis

Statistical package for the Social Sciences (SPSS) 23 was used for the data analysis. Descriptive statistics were calculated for all study variables. This includes the mean and standard deviation for continuous measures, counts and percentages for categorical variables. Comparison of means was performed using the non-parametric tests since the sample was not normally distributed. The Mann–Whitney test was used to compare between two means, whereas the Kruskal–Wallis test was performed to compare means three or more means. The Spearman correlation was used for linear correlation between continuous variables. Two forward linear regressions were carried out taking the girl’s age at menarche as the dependent variable and using variables that showed a *p* value < 0.05 in the bivariate analysis as independent variables. In the first model, the NICU admission due to any cause was taken as an independent variable, whereas in the second model, each cause for NICU admission was taken as an independent variable. Significance was set at a *p* < 0.05.

## Results

### Sample description

No significant difference was found between included and excluded girls in terms of sociodemographic characteristics, thus, eliminating the risk of selection bias (data not shown). Table 1 represents sociodemographic criteria and other characteristics of the girls. At birth, the mean gestational age was 37.63 ± 2.74 weeks (min = 26; max = 42), with a mean birth weight of 3032.08 ± 725.44 g, and a mean length of 46.82 ± 4.98 cm. the mean Apgar score at 1 min was 8.52 ± 1.43 (min = 0; max = 10), whereas the mean Apgar at 5 min was 9.56 ± 1.32 (min = 0; max = 10). It is noteworthy that 20 (6.8%) of the participants had an Apgar score at 1 min < 7, whereas 9 (3.1%) had an Apgar score at 5 min < 7. Moreover, 153 girls (51.7%) were born by C-section, whereas 143 (48.3%) were born by normal vaginal delivery. A preeclampsia during pregnancy took place in 7 mothers (2.4%). In addition, 96 girls (32.4%) were admitted to the NICU, with 62 (20.9%) premature and 19 (6.4%) SGA. The mean days of phototherapy was 1.01 ± 2.19 (min = 0; max = 11). We had 9 girls (3.0%) who needed hood oxygenation at birth, 17 (5.7%) intubated and ventilated, with 4 (1.4%) who received surfactant. During childhood, 42 girls (14.2%) developed asthma. The mean actual age of the participants was 15.57 ± 1.64 years (min = 12.01; max = 19.00), with a mean actual BMI of 21.31 ± 3.19 kg/m^2^ (min = 13.84; max = 37.60). All participants menstruated; the mean age of menarche was 12.39 ± 1.39 years, whereas the mean age of menarche in their mothers was 12.44 ± 1.29 years. Finally, 15 adolescents (5.1%) suffered from PCOS, with 70 (23.6%) girls having a problem with the regularity of their menstruations.

**Table 1:** Sociodemographic and other characteristics of the participants (N = 297)

Variable	Mean ± SD
Actual age (in years)	15.57 ± 1.64
Gestational age (in weeks)	37.63 ± 2.74
Birth weight (in grams)	3032.08 ± 725.44
Birth length (in cm)	46.82 ± 4.98
Father’s age at birth (in years)	35.87 ± 4.89
Mother’s age at birth (in years)	30.42 ± 4.82
Mother’s age at menarche (in years)	12.44 ± 1.29
Girls’ age at menarche (in years)	12.39 ± 1.39
Days of menstruation in girls	4.95 ± 1.10
Parity	1.91 ± 1.02
Apgar at 1 min	8.52 ± 1.43
Apgar at 5 min	9.56 ± 1.32
Actual Body Mass Index (in kg/m^2^)	21.31 ± 3.19
Days of phototherapy	1.01 ± 2.19

	N (%)
Delivery mode	
Vaginal	143 (48.3%)
Cesarean	153 (51.7%)
Admission to the NICU (yes)	96 (32.4%)
Small for gestational age (yes)	19 (6.4%)
Prematurity (yes)	62 (20.9%)
Breastfeeding (yes)	217 (73.3%)
Neonatal infection (yes)	54 (18.2%)
Maternal smoking during pregnancy (yes)	14 (4.7%)
Maternal smoking after pregnancy (yes)	79 (26.7%)
Preeclampsia (yes)	7 (2.4%)
Administration of glucocorticoids to the mother (yes)	25 (8.4%)
Administration of glucocorticoids to the newborn (yes)	2 (0.7%)
Ventilation	
No	270 (91.2%)
HOOD	9 (3.0%)
Synchronized Intermittent Mandatory Ventilation (SIMV)	17 (5.7%)
Administration of surfactant (yes)	4 (1.4%)
Anemia (yes)	19 (6.4%)
Hirsutism	
No	111 (37.5%)
Light	155 (52.4%)
Moderated	30 (10.1%)
Family history of prematurity (yes)	20 (6.8%)
Asthma during childhood (yes)	42 (14.2%)
Polycistic ovary syndrome (PCOS)	15 (5.1%)
Intake of oral contraceptives (yes)	9 (3.0%)
Problems in the regularity of the girl’s period (yes)	70 (23.6%)
Need to change pads during the night (yes)	22 (7.4%)
Presence of big clots during the bleeding (yes)	8 (2.7%)

### Bivariate analysis

The significant and non-significant bivariate analysis results of factors associated with the girl’s age at menarche are summarized in Tables 2 and 3.

**Table 2:** Significant results of bivariate analysis of factors associated with the girl’s age at menarche

Variable	Mean ± SD
Gestational age	
r	0.144
*p* value	*0.013*
Birth weight	
r	0.166
*p* value	*0.004*
Birth length	
r	0.186
*p* value	*0.021*
Mother’s age at menarche	
r	0.198
*p* value	*0.001*
Days of phototherapy	
r	− 0.224
*p* value	*< 0.001*
Admission to the NICU for any reason	
No	12.60 ± 1.44
Yes	11.95 ± 1.17
*p* value	*< 0.001*
Small for Gestational Age (SGA)	
No	12.45 ± 1.38
Yes	11.61 ± 1.31
*p* value	*0.011*
Prematurity	
No	12.53 ± 1.39
Yes	11.87 ± 1.27
*p* value	*0.001*
Neonatal infection	
No	12.51 ± 1.46
Yes	11.89 ± 0.87
*p* value	*< 0.001*
Maternal smoking during pregnancy	
No	12.41 ± 1.41
Yes	11.82 ± 0.77
*p* value	*0.042*
Preeclampsia during pregnancy	
No	12.42 ± 1.39
Yes	11.31 ± 0.98
*p* value	*0.037*
Administration of glucocorticoids to the mother during pregnancy	
No	12.48 ± 1.36
Yes	11.49 ± 1.38
*p* value	*0.001*
Asthma during childhood	
No	12.47 ± 1.40
Yes	11.89 ± 1.24
*p* value	*0.011*
Ventilation (HOOD or SIMV) + surfactant (yes)	
No	12.43 ± 1.43
Yes	12.06 ± 0.86
* p* value	0.06
Anemia	
No	12.48 ± 1.32
Yes	11.15 ± 1.82
*p* value	*0.005*

Numbers in italics indicate significant association; other variables that are not displayed in the table did not show any significance

**Table 3:** Multivariate analysis

Variable	Unstandardized beta		*p* value	95% confidence interval	
Model 1: Linear regression taking the girl’s age at menarche as the dependent variable and taking the NICU admission due to any cause as an independent variable					
Mother’s age at menarche	0.209		0.009	0.052	0.366
Gestational age	0.069		0.018	0.012	0.125
Asthma during childhood in the girl (yes vs no*)	− 0.526		0.020	− 0.969	− 0.083
Model 2: Linear regression taking the girl’s age at menarche as the dependent variable and taking each cause for NICU admission as an independent variable					
Number of days of phototherapy	− 0.128	< 0.001		− 0.198	− 0.059
Mother’s age at menarche	0.223	< 0.001		0.107	0.340
Preeclampsia during pregnancy (yes vs no*)	− 1.168	0.022		− 2.163	− 0.172
Asthma during childhood in the girl (yes vs no*)	− 0.504	0.024		− 0.941	0.068

Variables entered in model 1: Gestional age, mother’s age at menarche, maternal smoking during pregnancy and girl’s asthma later in life

*Reference group; Variables entered in model 2: Gestional age, mother’s age at menarche, number of days of phototherapy, small for gestational age, prematurity, materno-fetal infection, maternal smoking during pregnancy, high blood pressure in the mother during pregnancy, mother intake of corticosteroids during pregnancy, girl’s asthma later in life

A higher gestational age (r = 0.144), weight (r = 0.166) and height (r = 0.186) at birth and mother’s age at menarche (r = 0.198) were significantly but weakly associated with a higher girl’s age at menarche. In addition, a higher girl’s age at menarche was found in those who were not admitted to the NICU (12.60 vs 11.95), who were not SGA (12.45 vs 11.61), who were not premature (12.53 vs 11.87), who did not manifest a neonatal infection (12.51 vs 11.89), who came from mothers who did not smoke during pregnancy (12.41 vs 11.82), mothers who did not suffer from preeclampsia (12.42 vs 11.31) and mothers who did not take corticosteroids during pregnancy (12.48 v 11.49) compared to those who did not have these conditions.

### Multivariable analysis

The results of a first stepwise linear regression taking the girl’s age at menarche as the dependent variable and taking the NICU admission due to any cause as an independent variable, showed that asthma later in life (Beta = − 0.526) was significantly associated with lower age at menarche in girls, whereas a higher mother’s age at menarche (Beta = 0.209) and a higher gestational age (Beta = 0.069) were significantly associated with higher age at menarche in girls (Table 3, Model 1).

The results of a second stepwise linear regression taking the girl’s age at menarche as the dependent variable and taking each cause of NICU admission as an independent variable, showed that a higher mother’s age at menarche (Beta = 0.223) was significantly associated with higher age at menarche in girls, whereas a higher number of days of phototherapy (Beta = − 0.128), a preeclampsia in the mother during pregnancy (Beta = − 1.168) and asthma later in life in the girl (Beta = − 0.504) were significantly associated with a lower age at menarche in girls (Table 3, Model 2).

## Discussion

Our results showed that a higher gestational age in girls at birth and a higher menarcheal age in the mothers were significantly correlated to a higher age at menarche in girls. However, girls whose mothers suffered from preeclampsia during their pregnancy, those who were exposed to phototherapy during the neonatal period and those who suffered from asthma during their childhood, developed their menarche earlier than those who didn’t present these conditions. Finally, neonatal respiratory distress and being small for gestational age were not significantly associated to menarcheal age.

### Childhood asthma

Our study showed that asthma during childhood was significantly associated to a more lower age at menarche. This disagrees with the studies demonstrating that asthma by itself, as well as its treatment by inhaled corticosteroids, lead both to a pubertal delay [32, 33, 49]. In the literature, an association was revealed between respiratory distress at birth, even in infants born at term, and the high risk to develop asthma later on [50]. Since asthma in our study led to a lower age at menarche in girls, we hypothesized that this precocity may be influenced by respiratory morbidities at birth and the need of oxygen therapy. However, the relationship between neonatal respiratory distress and the timing of menarche was not significant in our study. In fact, by grouping together the necessity of ventilation and the administration of surfactant to relate them to the menarcheal age, the result of the bivariate analysis was very close to be significant. This may be due to the small number of infants who needed an oxygen therapy at birth during this period in the NICU of our hospital. More studies are recommended to try to find a significant relationship between lower age at menarche and neonatal respiratory distress, especially that this subject is not discussed in the literature till now. In addition, other studies showed an association between prematurity and the development of asthma later on [51, 52].

### Mean age of menarche in girls

The girls’ mean age of menarche in our study was 12.39 years, which is accelerated by 0.11 years compared to a study made on adolescent Lebanese girls in 2012, having a mean age at menarche of 12.5 years [53]. Comparing our results to other countries, menarche in Lebanese Girls was delayed by 0.29 years compared to Saudi Arabia [54], while earlier 0.11 years than France [55] and 0.01 years than Italy [56]. Noting that premature girls in our study had a mean age at menarche of 11.87 years, while it was 12.53 years in girls born at term.

### The effect of maternal menarcheal age

In our study, we demonstrated that a higher maternal age at menarche was associated with a higher age at menarche in their descendants. This is in agreement with the studies analyzing the association between these two factors, showing the strong correlation between them [20, 21, 57]. However, our result was not affected by the BMI of the girls, in contradiction to the studies demonstrating that maternal age at menarche can only predict the age of the first menstruation in non-obese girls [22]. This proven relationship highlights the importance of the possible influence of genetic factors, which may determine the variation of the timing of menarche and puberty in girls in around 50–80% of the time [18, 19, 21]. The mean age of mothers’ menarche in our study was 12.44 years, delayed by 0.05 years than that of the girls, which can be explained by the trend toward an earlier puberty during these years, which stabilized recently [58].

### The effect of phototherapy exposure

Phototherapy exposure during the neonatal period was significantly associated with a lower age at menarche in adolescents, and this depended on the number of days of exposure. This is described for the first time in the literature, and no study till now has analyzed the relationship between phototherapy and the timing of menarche or puberty. Given that most of the premature babies born at less than 35 weeks will increase their plasma bilirubin levels and develop a neonatal jaundice, and that these infants present a high risk to develop cerebral lesions associated with hyperbilirubinemia [59], these premature girls will be more exposed to phototherapy as treatment or even as prevention [59].

### Preeclampsia during pregnancy

Our results showed that preeclampsia in mothers during pregnancy was significantly associated with a younger age at menarche in their daughters. This is in disagreement with the studies showing that this association was not significant after adjustment over confounding factors [22, 23, 57, 60]. As already mentioned, hypertension constitutes a major cause of intrauterine growth restriction and may result in a deficient metabolic state at birth, which may then lead to a lower age at menarche [25].

### Birth weight, length and SGA

An association in the bivariate, but not the multivariable, analysis was demonstrated between a higher age at menarche and the increase in birth weight and length, as well as in girls who were not SGA, in opposite to the studies demonstrating that menarche took place earlier in SGA girls comparing to those born appropriate for gestational age [28]. The non-significance of this association in our multivariate analysis may be due to the small number of SGA infants in our study. A larger sample may lead to more significant results, proving the existence of a relationship between a lower age at menarche and the adolescents born SGA.

### Glucocorticoids administration during pregnancy

In general, glucocorticoids administered to the mother during at-risk pregnancies contribute to fetal lungs maturation, and reduce the incidence of neonatal respiratory distress syndrome or any respiratory distress at birth [23]. However, studies associated the fetal corticoids exposure to a disruption in endocrine functions and in reproduction later on in girls, leading to a delay in puberty [22, 23]. Nevertheless, the bivariate, but not the multivariable, analysis in our study showed an advanced age of menarche in girls who were not exposed to glucocorticoids during the antenatal period. This precocity may be due to the fact that premature girls are the most at risk to have received doses of glucocorticoids during the fetal life, and that a significant relation between prematurity and menarcheal precocity may be attenuated under the effect of corticoids administration. It’s noteworthy that in our study, only 25 mothers received glucocorticoids doses (8.4% only), and so a larger sample could make the results more significant.

### Prematurity

Prematurity as an independent factor was not found to be significantly associated with the age of the first menstruations, unlike the bivariate analysis where it has been demonstrated that premature girls manifest their menarche at a younger age comparing to those born at term. This is in agreement with the studies showing that a lower age at menarche takes place in premature girls comparing to those born at term [26, 61]. However, in the literature revue done by Evelyn James and al. in 2018, most of the studies did not show any association between gestational age and the timing of menarche, concluding the absence of association between prematurity and lower age at menarche [26].

### Limitations

Our study has many limitations. The sample size is small and doesn’t represent all the Lebanese population. In fact, the number of SGA infants, of newborns needing an oxygen therapy at birth, and of mothers who received doses of glucocorticoids during pregnancy is much reduced. A larger sample is needed to obtain more significant results, and to empower the correlation between these factors and the menarcheal timing. In addition, a selection bias is possible, since many girls were lost during these years, and the data was collected from only one medical center, so a multi-centric study is recommended to be able to generalize the results; however this bias was reduced by the non-significant difference between included and excluded girls. In addition, this is a cross sectional study with retrospective results, so it has a low level of evidence and cannot infer causality. A possible recall bias may be present due to the retrospective aspect of our study, leading to an underestimation or an overestimation of the effects of some risk factors (days of breastfeeding, number of cigarettes during pregnancy, weight and height at menarche in girls, asthma during childhood). Prospective studies which avoid the recall bias should ameliorate the precision of our results. Finally, an information bias may also be possible since the use of a questionnaire led by phone in a young population or even in parents may not provide accurate answers: A deficit in remembering, evaluating symptoms, and in the accuracy of information may be possible, such as the regularity and the abundance of menstruations, hirsutism, the exact age of menarche in mothers and in girls, actual weight and height. However, our methodology is similar to other cross sectional studies, and may be considered as a pilot study that will guide further studies on a larger scale.

## Conclusion

The timing of menarche seems to be affected by many factors in this population of girls born in Lebanon.

Our results showed that a higher menarcheal age in mothers, and a higher gestational age in girls at birth were both significantly associated with a higher age at menarche in girls. However, girls whose mothers suffered from preeclampsia during their pregnancy, those who were exposed to phototherapy during the neonatal period and those who suffered from asthma during their childhood, developed an earlier menarche than those who didn’t present these conditions.

Given that precocious menarche is associated with many diseases later on in life, physicians should not disregard these factors and should search for pubertal signs earlier in girls at risk, and then judge on a case-by-case basis if an intervention is needed or not.

More extended studies are needed to confirm our preliminary results, and a bigger sample may confirm a significant relationship between SGA, respiratory distress and need of oxygen therapy at birth with a precocious menarche.

## Supplementary information

**Additional file 1.** The questionnaire developed for this study is provided as Additional file 1.

## Data Availability

There is no public access to all data generated or analyzed during this study to preserve the privacy of the identities of the individuals. The dataset that supports the conclusions is available to the corresponding author upon request.

## Abbreviations

NICU: Neonatal intensive care unit
SGA: Small for gestational age
SPSS: Statistical package for the Social Sciences
