# Supplementary material for: Association between factors related to the pregnancy, neonatal period, and later complications (especially asthma) and menarcheal age in a sample of Lebanese girls
Source: BMC Womens Health. 2020 Oct 16;20:236. doi: 10.1186/s12905-020-01101-7 (PMC7565354; doi:10.1186/s12905-020-01101-7)
Supplement: Supplementary file 1 — Additional file 1. The questionnaire developed for this study is provided as Additional file 1. [file 12905_2020_1101_MOESM1_ESM.docx]

**Additional file 1- Questionnaire**

**Oral consent given:**

- Yes. Continue the survey.
- No. Stop the survey.

**I. Data collected from the girls’ medical files:**

A. Generalities:

1) Name:

2) Family name:

3) Father’s name:

4) ID number:

5) Date of birth:

6) Phone number:

**B. At birth:**

1) Admission to the NICU:

o No

o Yes

If yes:

i. Admission date:

ii. Discharge date:

iii. Death:

o Yes Date:

o No

2) Gestational age:

3) Prematurity:

o Yes

o No

4) Mode of delivery:

o Normal vaginal delivery

o C-section

5) Weight at birth:

6) Length at birth:

7) Head circumference at birth:

8) Parity:

9) Apgar score:

- At 1 minute:
- At 5 minutes:

10) Preeclampsia in the mother:

o Yes

o No

11) Administration of glucocorticoids to the mother during pregnancy:

o Yes

o No

12) Neonatal infection:

o Yes Duration of antibiotics (days):

o No

13) Respiratory distress at birth:

o Yes

o No

14) Necessity of ventilation:

o Yes

o No

i. If yes type of ventilation:

ii. Si yes, days of ventilation (Days):

15) Surfactant administration:

o Yes

o No

16) Administration of glucocorticoids to the girl:

o Yes Days of corticoids (days):

o No

17) Phototherapy:

o Yes days of phototherapy:

o No

**II. Questionnaire lead over the phone:**

**A. With the girls:**

1) Actual age:

2) Actual weight:

3) Actual height:

4) Age at menarche:

5) Weight at menarche:

6) Height at menarche:

7) Regularity of menstruations’ cycles:

o Yes

o No

8) Abundancy of bleeding:

i. Interval of changing tampons/pads:

o < 3 hours

o > 3 hours

ii. Number of tampons/pads per cycle:

o < 21 tampons/pads

o > 21 tampons/pads

iii. Need to change at night:

o Yes

o No

iv. Presence of big clots:

o Yes

o No

v. Presence of anemia:

o Yes

o No

9) Days of bleeding:

10) Diagnosis of a polycystic ovary syndrome:

o Yes

o No

11) Treatment with oral contraceptives:

o Yes

o No

12) Presence of hirsutism:

o No (Score <8)

o Light (Score between 8 and 15)

o Moderate (Score between 16 and 25)

o Severe (Score >25)

13) Smoking in girls:

o Yes

o No

If yes:

i. Cigarettes:

o Yes Number of cigarettes per day:

o No

ii. Water pipe:

o Yes Number of heads per week:

o No

iii. Cigars:

o Yes Number of Cigars per week:

o No

14) Passive smoking:

o Yes Number of smokers inside the house:

Frequency of exposure in cafes and restaurants per week:

o No

**B. With the mothers:**

1) Mother’s date of birth:

2) Father’s date of birth:

3) Age at menarche in mothers:

4) Breastfeeding:

o Yes Duration (months):

o No

5) Smoking during pregnancy:

o Yes

o No

If yes:

i. Cigarettes:

o Yes Number of cigarettes per day:

o No

ii. Water pipe:

o Yes Number of heads per week:

o No

iii. Cigars:

o Yes Number of cigars per week:

o No

6) Alcohol during pregnancy:

o Yes

o No

If yes type of alcohol:

If yes number of glasses per week:

7) Smoking after pregnancy:

o Yes

o No

If yes:

iv. Cigarettes:

o Yes Number of cigarettes per day:

o No

v. Water pipe:

o Yes Number of heads per week:

o No

vi. Cigars:

o Yes Number of cigars per week:

o No

8) Girl’s asthma during childhood:

o Yes

o No

9) Any familial history of prematurity:

o Yes

o No

10) Presence of any pathology during the girl’s life

o Yes

o No

If yes: Necessity to take any medication

o Yes Name of medication:

o No
